# EDITORS’ INTRODUCTION: Transgender Health Equity and the Law

**DOI:** 10.1017/jme.2022.83

**Published:** 2022

**Authors:** Heather Walter-McCabe, Alexander Chen

**Affiliations:** 1. WAYNE STATE UNIVERSITY, DETROIT, MI, USA,; 2. HARVARD UNIVERSITY, CAMBRIDGE, MA, USA.

**Keywords:** LGBTQ Health Equity, Transgender, Public Health, Gender Affirming Care, Structural Determinants of Health

## Abstract

The sheer gamut of issues impacting transgender health equity may seem overwhelming. This article seeks to introduce readers to the breadth of topics addressed in this symposium edition, exemplifying that transgender health equity is a global issue that demands an interdisciplinary approach.

This special edition of the *Journal of Law, Medicine & Ethics* comes at a critical time — a time when legislators in multiple states across the country have proposed the highest number of anti-LGBTQ+ bills in recent history.^1^ Transgender communities, here defined inclusively to include transgender, nonbinary, genderqueer and all those whose gender identity does not match their sex assigned at birth, have been particularly affected. Lawmakers in 26 states introduced bills to ban transgender youth from accessing health care; health care which the American Medical Association, the American Academy of Pediatrics, the American Psychiatric Association, and the American Academy of Child & Adolescent Psychiatry all recognize as medically appropriate care.^2^ Transgender youths’ ability to participate in athletics, ability to use facilities such as bathrooms and locker rooms according to their gender identity, and ability to be raised in a supportive home with their families are also being debated — and restricted — in the legislative and public sphere.^3^

These laws and attendant public discourse negatively impact transgender people by increasing stigma and discrimination, as well as in some cases by specifically eliminating access to gender-affirming health care services. Transgender people already experience health inequities at a disparate rate compared to their cisgender peers, including increased rates of mental health disorders, substance use disorders, sexual and physical violence, and sexually transmitted infections. Transgender people, particularly transgender women, experience violent injury and death at an disturbingly disparate rate.^4^ Suicidality rates are also alarming, with the most recent survey of transgender adults in the United States finding a suicide attempt rate nearly nine times that of the general population,^5^ and nearly 35% of transgender youth reporting a suicide attempt in a 2017 Centers for Disease Control study.

By contrast, support and affirmation of transgender people is associated with positive health outcomes.^6^ For example, a 2021 survey of transgender youth by The Trevor Foundation found that youth who had access to social support and gender affirmation — including use of gender-affirming pronouns, clothing, and access to legal name and gender changes — were significantly less likely to attempt suicide.^7^ Other studies have examined the impact of access to affirmative medical treatment. A 2020 study compared the psychological well-being of transgender adolescents before and after receiving puberty suppression with their cisgender peers and transgender youth who had been referred to a gender affirming clinic but had not received puberty suppression. The study found that while transgender youth prior to treatment generally reported more emotional and behavioral issues and self-harm or suicidal ideation than their cisgender peers, those transgender youth who received affirming medical care inclusive of puberty suppression reported similar or better psychological well-being than their cisgender peers, and better psychological well-being than their transgender peers who received care through a gender affirming clinic without receiving puberty suppression.^8^ Another study found that when adolescents who desired puberty suppression were provided with this treatment, there was a significant association with lowered suicidal ideation across the person’s lifetime.^9^ Short-term studies of transgender adolescents and young adults within the first year of receiving puberty suppression or gender-affirming hormones show similar outcomes.^10^

As this brief overview of the issues illustrates, law and policy can have a profound impact on health outcomes for transgender people individually and the health of transgender populations broadly. Beyond the health impacts described above, housing discrimination, workplace discrimination, access to public accommodations, and other social determinants of health are all implicated when considering transgender health equity. This symposium edition provides an opportunity for scholars working in this field to shed light on particular areas of inquiry.

Public health law scholars study law as an important additional tool to impact public health issues.^11^ Transgender health equity, like other public health issues, can be positively or negatively impacted by state and federal laws. A growing evidence base supports the intuitive proposition that laws which support inclusion and fight discrimination have a positive impact on health outcomes for transgender people. For example, a study by Baumle et al.^12^ examined the impact of federal employment laws providing protections for sexual orientation and gender identity in states which fail to provide these protections, and found that complaints filed before the federal Equal Employment Opportunity Commission in such states were of a more serious nature and featured higher incidents of harassment than complaints filed in states with state-based protections. The researchers concluded that their findings “provide evidence that legal and sociopolitical context are likely shaping the discriminatory experiences and disputing behaviors of LGBT individuals.”^13^

Researchers have also examined the impact of discriminatory experiences on health outcomes for LGBTQ+ populations. Early research by Ilan Meyer described the concept of minority stress theory, which posits that discrimination and stigma bring about unique stressors which ultimately negative impacts both physical and mental health outcomes.^14^ Other researchers have subsequently studied the impact of policy and associated stigma and discrimination on LGBTQ+ populations in a variety of situations, including youth suicide rates and local level ecological factors,^15^ youth suicide attempts and anti-bullying policy,^16^ and psychiatric issues and state-level measures of LGBTQ+ related policy.^17^ While earlier research tended to focus primarily on lesbian, gay, and bisexual individuals, researchers are increasingly centering transgender and gender diverse populations as they examine the impact of law and policy on health outcomes.^18^ Wesp and colleagues created a new conceptual framework, the Intersectionality Research for Transgender Health Justice (IRTHJ) Framework, for examining structural forms of injustice, including policies, and their impact on transgender populations’ health.^19^ Reisner and colleagues specifically examined how legal protections, and lack thereof, for transgender and gender minority populations impacted their ability to access health care.^20^ Legal scholars are actively working to understand how recent legal cases, some protective like *Obergefell v. Hodges* (marriage equality) and *Bostock v. Clayton County* (employment discrimination),^21^ and some regressive like *Dobbs v. Jackson Women’s Health Organization* (abortion access) are impacting the lived experiences of transgender people and their experiences of stigma and discrimination.^22^ Other topics are currently in litigation and, though scholars will most certainly be analyzing the impact of the cases, are so new that there is little legal scholarship available. Skilled litigators are developing our understanding not just by developing new precedent, but also by educating the public about the issues implicated in recent litigation.^23^

Along with litigation and state level laws, federal regulatory regimes play an important role in access to care and health equity. Since the passage of the Patient Protection and Affordable Care Act (ACA)^24^, the law’s antidiscrimination provision, known as Section 1557, has been caught in a regulatory tug-of-war between successive presidential administrations, which have vigorously disagreed on whether that provision’s bar on sex discrimination should be interpreted to include discrimination on the basis of sexual orientation or gender identity — including requiring public and private insurance providers to cover gender-affirming care.^25^ At the time of publication, the Biden administration has published a new proposed rule regarding its interpretation of Section 1557, which would reinstitute provisions forbidding discrimination on the basis of gender identity and requiring covered entities to provide gender-affirming care. This interpretation of the provision’s scope is consistent with the Supreme Court’s ruling in *Bostock v. Clayton County*, 140 S. Ct. 1731 (2020), that the federal employment law Title VII’s analogous bar on sex discrimination also encompassed homosexuality and transgender status. If the final rule includes this provision, it does not preclude court cases challenging it, but would be a positive movement towards more inclusive medical care for transgender communities.

As this brief overview of the issues illustrates, law and policy can have a profound impact on health outcomes for transgender people individually and the health of transgender populations broadly. Beyond the health impacts described above, housing discrimination, workplace discrimination, access to public accommodations, and other social determinants of health are all implicated when considering transgender health equity. This symposium edition provides an opportunity for scholars working in this field to shed light on particular areas of inquiry. Figure 1 provides a glossary of terms which might be of assistance as you read the articles.

**Figure 1:**
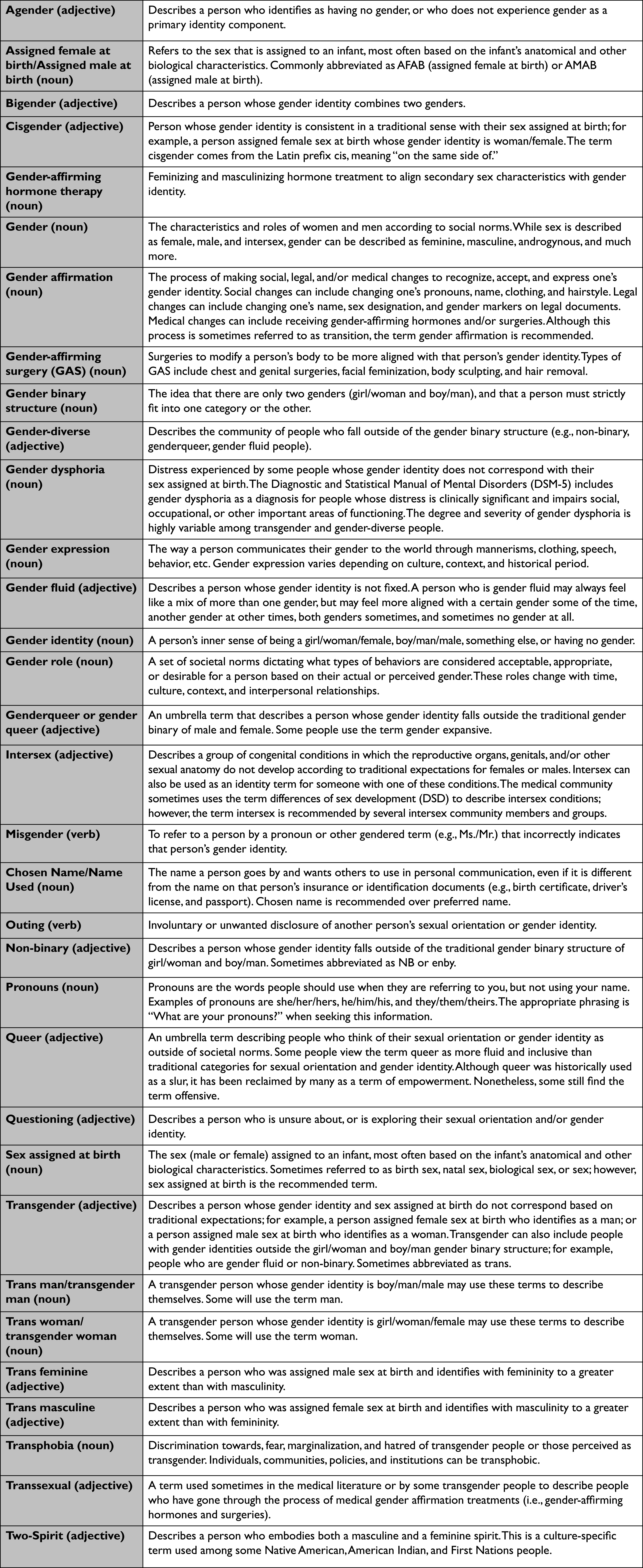
Glossary of Terms These terms and definitions are taken from the National LGBTQIA+ Health Education Center, a Program of the Fenway Institute. For a full list of terms regarding sexuality and gender identity, please see https://www.lgbtqiahealtheducation.org/publication/lgbtqia-glossary-of-terms-for-health-care-teams/

We begin with an article by Aoife O’Connor and colleagues which puts the topic of transgender health equity in the context of global health.^26^ The article provides an overview and analysis of international law and human rights as it relates to transgender communities. The authors conclude that “unencumbered legal recognition of gender identity and expression under international human rights law should be central to advancing global health.”

Florence Ashley examines rights and equality through the legal and social landscape of conversion practices, “attempts to alter, discourage, or suppress a person’s gender identity and/or desired gender presentation, including by delaying or preventing gender transition.”^27^ She walks the reader through a description of transgender conversion practices and research regarding its impact, examining the legal landscape with a particular emphasis on the analysis of expressive equality. She examines concepts of professional responsibility for both medical and legal practitioners and ultimately makes the case that those who wish to practice conversion therapy, not those who oppose it, bear the burden of justifying the practice.

The next two articles explore issues of health care access and coverage for transgender communities. Poteat and Simmons write about their innovative research project, TRANSforming the Carolinas, where they study the impact of multiple identities and structural disparities on transgender communities in North and South Carolina.^28^ Their research, designed to eliminate health disparities for transgender people of color living with HIV, provides insight into how policy impacts this population, both through policy analysis and through input from the community itself. Importantly, this study emphasizes the inclusion of transgender people and their self-identified needs in structuring recommendations for policy and practice interventions.

Baker and Restar also examine access to care in their article studying private insurance utilization and costs for gender-affirming care.^29^ Their study seeks to fill gaps in the literature regarding gender-affirming care as insurers see increased requests for these important services. Their data can help inform policymakers as states continue to determine when and if gender-affirming care will be provided. It can also serve to inform federal policymakers as they promulgate a final rule under section 1557 of the Affordable Care Act, discussed above, and other policies increasing access to services for transgender people.

Kukura examines a specific topic of concern under current policy circumstances: transgender pregnancy.^30^ Kukura discusses barriers to transgender people assigned female at birth and access to culturally appropriate pregnancy and postpartum care. As she aptly writes, this article is important “as scholars move beyond questions asked in some early literature about *whether* trans people should get pregnant to focus instead on how to best meet the needs of [transgender] people during pregnancy and childbirth.”

A final article from Kinney and colleagues surveys the current legislative anti-transgender backlash and offers a gender equity impact tool for use in creating more equitable and transgender inclusive policies going forward.^31^ The proposed tool, designed for policymakers, advocates, and community members, and building on the work of those in the field of diversity, equity, and inclusion, centers insights from transgender community members through use of a community advisory board (CAB). The CAB, as the authors write, is at the heart of the tool and represents “an ongoing commitment *with* and *by* community for [transgender] inclusion and equity.” We hope that this tool provides interested readers a tangible roadmap to working toward creating more equitable policies and societal structures to move the needle on transgender health equity.

This edition concludes with an essay by pioneering transgender advocate Jamison Green, a former President of the World Professional Association for Transgender Health.^32^ Dr. Green provides a revelatory personal historical perspective on the decades-long struggle of transgender people to be treated with human dignity and professional competence by providers and the health care system. As Dr. Green’s poignant essay showcases, transgender communities themselves are the true experts on the issues impacting them. Researchers have long recognized the importance of community participation in attaining better health policy outcomes.^33^ To that end, we sought to ensure that all articles involved members of the community as authors, co-authors, and/or reviewers. We encourage those who read this symposium edition and want to work to create more inclusive policy to ensure that part of your process is inviting transgender community members to the table and meaningfully engaging with them.

The sheer gamut of issues impacting transgender health equity may seem overwhelming. As the breadth of topics addressed in this symposium exemplifies, transgender health equity is a global issue that demands an interdisciplinary approach. Even the variation in the terms that are used to describe the community within these pages testifies to the range of life experiences — and corresponding health equity challenges — borne by transgender people. However, the papers in this symposium also demonstrate the profound and cumulative benefits of incremental policy changes and advancements in learning that have steadily improved health outcomes over time. Whether the reader seeks to garner a beginning understanding of transgender health equity, or is a scholar, practitioner, or policymaker already involved in this work, we hope this symposium provides an enhanced understanding of the public health imperative of working to improve health equity for transgender communities, and how law and policy can be used as a tool to do so.

## Note

The authors have no conflicts to disclose.
